# Application of metabolomics in viral pneumonia treatment with traditional Chinese medicine

**DOI:** 10.1186/s13020-019-0229-x

**Published:** 2019-03-12

**Authors:** Lili Lin, Hua Yan, Jiabin Chen, Huihui Xie, Linxiu Peng, Tong Xie, Xia Zhao, Shouchuan Wang, Jinjun Shan

**Affiliations:** 1 Jiangsu Key Laboratory of Pediatric Respiratory Disease, Institute of Pediatrics, Affiliated Hospital of Nanjing University of Chinese Medicine, No. 138, Xianlin Avenue, Qixia District, Nanjing, 210023 China; 2 Medical Metabolomics Center, Nanjing University of Chinese Medicine, Nanjing, 210023 China; 3 The First Affiliated Hospital of Zhejiang, Chinese Medical University, Hangzhou, 310006 China; 4 School of Holistic Integrative Medicine, Nanjing University of Chinese Medicine, Nanjing, 210023 China

**Keywords:** TCM, Treatment, Metabolomics, Virus, Pneumonia

## Abstract

Nowadays, traditional Chinese medicines (TCMs) have been reported to provide reliable therapies for viral pneumonia, but the therapeutic mechanism remains unknown. As a systemic approach, metabolomics provides an opportunity to clarify the action mechanism of TCMs, TCM syndromes or after TCM treatment. This review aims to provide the metabolomics evidence available on TCM-based therapeutic measures against viral pneumonia. Metabolomics has been gradually applied to the efficacy evaluation of TCMs in treatment of viral pneumonia and the metabolomics analysis exhibits a systemic metabolic shift in lipid, amino acids, and energy metabolism. Currently, most studies of TCM in treatment of viral pneumonia are untargeted metabolomics and further validations on targeted metabolomics should be carried out together with molecular biology technologies.

## Introduction

Pneumonia is the world’s leading cause of death in young children and elderly people. Many pathogens are associated with pneumonia, and now attention is turning to the importance of viruses as pathogens [1]. In western medicine, viral pneumonia is defined as a disease in which there are gas exchange abnormalities at the alveolar level accompanied by inflammation of the lung parenchyma [2]. While, in the theoretical system of traditional Chinese medicine (TCM), the etiologies of this disease are classified into external and internal causes. Exterior pathogenic “wind and heat” invades the weak lung (interior cause) through the skin, mouth or nose, causing lung qi obstruction and stagnation. The main pathological products are phlegm, heat and blood stasis, manifesting with fever, cough, dyspnea, wheezing, nasal flaring, etc. The basic treatment principles are to regulate the lung qi, resolve phlegm, and relieve cough and dyspnea. The TCM prescriptions is composed of various kinds of medicinal plants, animals and minerals in the form of oral liquid, powder and granules.

According to the TCM treatment principle, traditional Chinese medicines (TCMs) have been reported to cure viral pneumonia in lots of ancient literature and modern research [3–7]. Our research group has been engaged in TCM treatment of children with viral pneumonia for nearly 20 years. Jinxin oral liquid (JOL), modified from ma-xing-shi-gan decotion, is proved to have good treatment effects both in clinic and experimental studies [8]. Although, TCMs in treatment of viral pneumonia have achieved certain therapeutic effects, the mechanism of TCMs remains unclear.

Omics technologies, which system biology bring, are valuable tools for TCM research. Metabolomics belongs to system biology and omics, is a new logical approach to search for functional small-molecules to evaluate the pharmacological effect of TCMs [9–11]. In recent years, our research group has conducted a series of metabolomics research to examine the effect of TCMs in treatment of viral pneumonia [12–15]. In this review, we try to do the summary of recent metabolomics research and seek more evidences for the reliability of TCM in treatment of in viral pneumonia.

## Traditional Chinese Medicines (TCMs) and its application in viral pneumonia

TCMs have been reported to provide reliable therapy for almost 2500 years. Nowadays, TCMs are widely used for prevention and treatment of viral pneumonia in China and many other Asian countries [16]. The most commonly used anti-virus substances in TCMs are plant elements, such as root, rhizome, leaf, flower, fruit, seed, etc. Other non-botanic substances including animal and mineral products are also utilized [17]. Anti-virus TCM formulas originate from the profound experiences of practitioners with understanding of ancient Chinese medicine theories, and the methods of application have been passed down through oral history.

In recent research, TCMs were reported to boost body resistance against virus by modulating the immune response [17–20]. Many viruses have established sophisticated mechanisms to interact with the host immune system. TCMs have been shown to enhance antibody production, T cell proliferation, the expression of antigen specific CD4 + and CD8 + responses, and increase of the titers of IgG1, IgG2, and IgA. Along with the function to increase levels of Th1 type cytokine secretion and activation of alveolar macrophages, suppress Th2/Th17-responses, and maintain the balance between Th1 and Th2/Th17 cells for the prevention of viral pneumonia [21].

In addition, an increasing number of TCMs-derived active compounds, TCM herbs and formulas with direct antiviral activity are garnering evidence of experimental efficacy. TCMs can effectively inhibit viral attachment and cell internalization, in order to minimize the viral spread and replication. According to the literature and our previous research, resveratrol and baicalin exhibited certain antiviral activity against viruses in both plaque assay and mouse models [22]. Taking Jin Xin oral liquid and Qing Fei oral liquid as examples of patent medicines [8], they have been proved to inhibit inflammation, reduce viral replication and viral load in the lung tissues. Along with direct antiviral activity, TCMs could be beneficial in preventing viral infection due to its immune-modulatory effect, producing of IFN- and TNF-. Both IFN- and TNF- belong to type I interferon (IFN), which contribute to the innate immunity against viral infection. More attention should be paid to type I interferon (IFN) signaling pathway, for its good efficacy in triggering multiple antiviral mechanisms and synergizing with IFN in promoting antiviral activities [23].

## Metabolomics applications in precision medicine

**Fig. 1:**
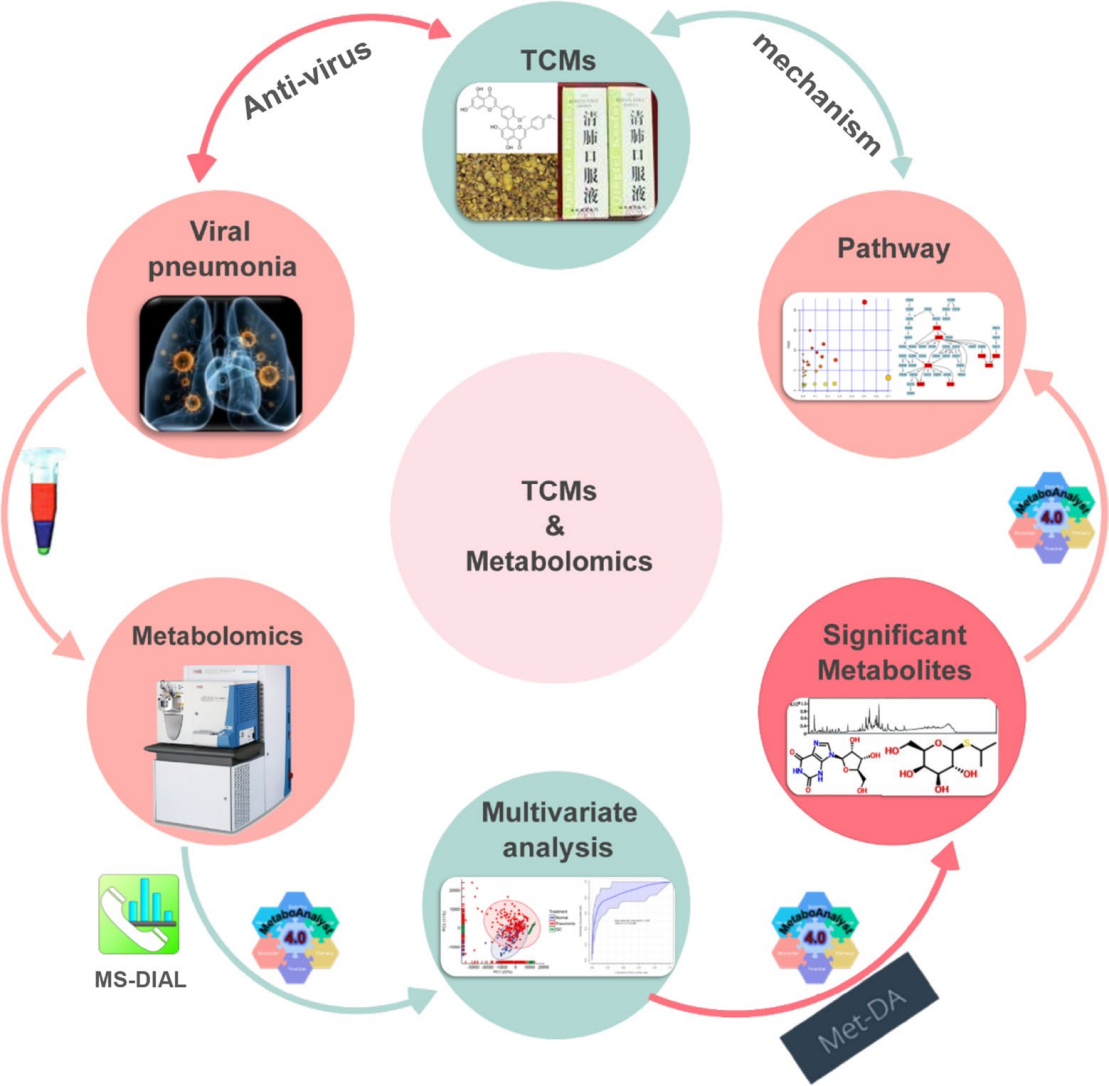
Workflows of metabolomics of TCMs in treatment of viral pneumonia. MS-DIAL, MetaboAnalyst 4.0 (https://www.metaboanalyst.ca) and MET-DA (http://metda.fiehnlab.ucdavis.edu) are metabolomics data analysis softwares or platforms

Metabolomics is the systematic study of the metabolic profile that specific cellular processes leave behind, including cells, tissues, and body fluids [24]. The metabolic profile provides a quantifiable readout of biochemical state from normal physiology to diverse pathophysiologies in a manner that is often not obvious from gene or protein levels [25]. Mass spectrometry (MS) combined with softwares or platforms (Fig. 1), such as MetaboAnalyst 4.0 (https://www.metaboanalyst.ca) [26], XCMS [27] and MS-DIAL [28], are effective tools for metabolite separation and collecting the information of the molecular composition of samples [29, 30], which will greatly facilitate the interpretation of the metabolomics data.

Increasing results have highlighted the usefulness of metabolomics as a promising laboratory tool for discriminating different chronic lymphocytic leukemia molecular subgroups [31] and discovering biomarkers for coronary artery disease progression [32]. The application of metabolomics to the prediction of the specific patient response to drug treatments is termed pharmacometabolomics, it is more closely associated to a patient´s pharmacological phenotype and could be more informative than genomic or proteomic data when trying to understand the mechanisms of inter-patient variability in response to drug therapy [33–35].

Examples that have emerged in the last few years demonstrating the potential of metabolomics tools and data in preclinical and clinical development. The study of metabolomics can potentially provide useful information for the diagnosis and prognosis of patients as well as for predicting pharmacological responses to specific interventions. Furthermore, specific metabolic signatures occur after drug treatment, thus providing information from pathways targeted or affected by drug therapy [36]. Thus, metabolomics, as a kind of logical strategy, is needed to achieve the goal of precision medicine.

## Metabolomics from precision medicine to viral pneumonia

Many microorganisms are associated with pneumonia, and now attention is turning to the importance of viruses as pathogens [1]. The most commonly identified agents of viral pneumonia in children were respiratory syncytial virus (RSV, 11%), influenza viruses (IFV, 10%), parainfluenza viruses (PIV, 8%) and adenovirus (ADV, 3%) [37, 38]. With a full set of tests in adults, findings of three reports suggested that a third of adult cases of pneumonia were associated with viral infection [39–41]. Similar to findings of pediatric studies, viral pneumonia in adults were noted with IFV (8%), RSV (3%), PIV (2%), and ADV (2%) [42, 43]. Nowadays, probably the clinical bottleneck for viral pneumonia is an inappropriately low level of clinical suspicion and corresponding lack of laboratory testing and effective anti-viral treatment. In cases of viral pneumonia where influenza A or B are thought to be causative agents, patients may benefit from treatment with oseltamivir or zanamivir [1]. For RSV pneumonia, RSV has no direct acting treatments. Ribavirin has been shown to exhibit potent activity against RSV in vivo and in vitro, however comparisons of the results of both animal and cell testing in humans have not yet been completed [44–46].

**Fig. 2:**
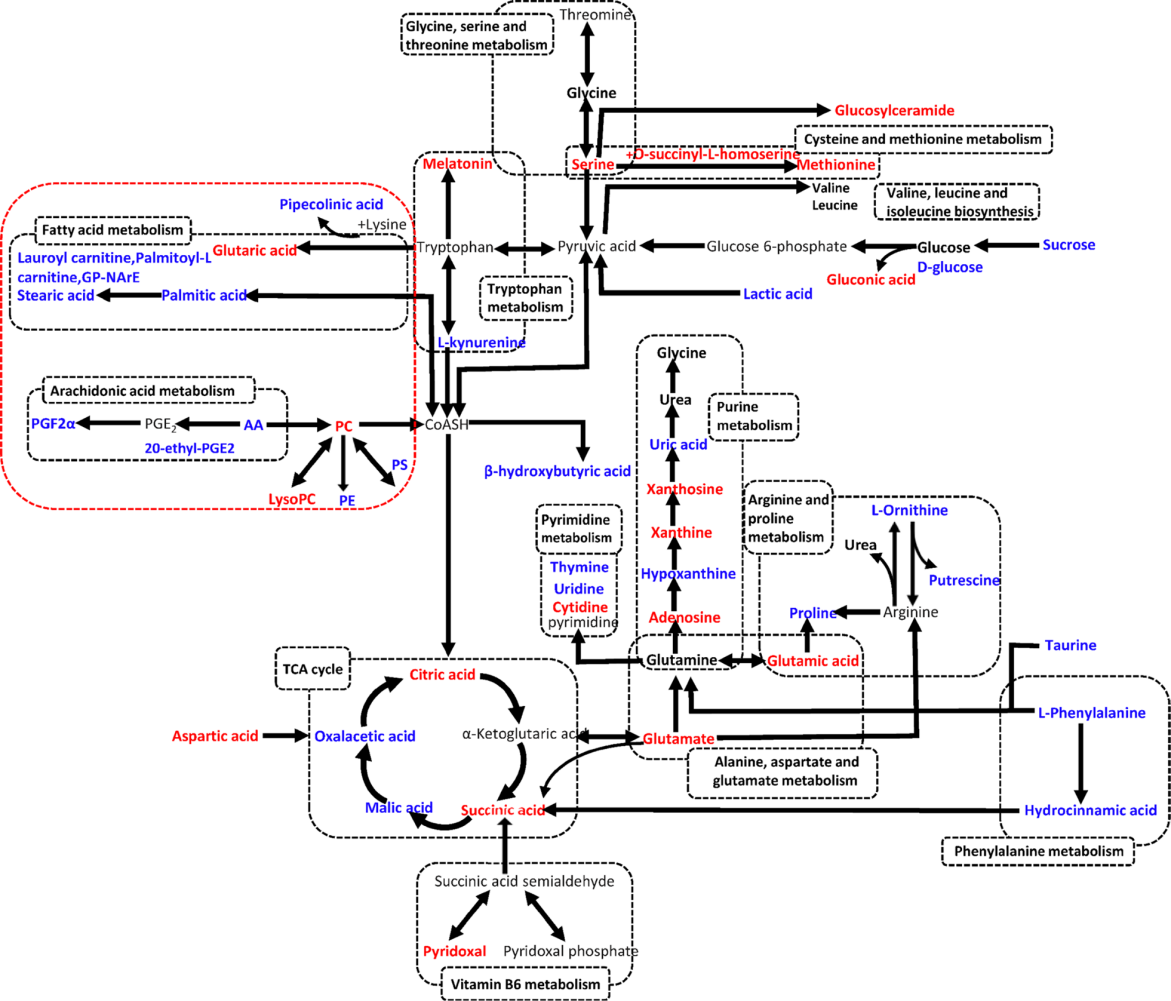
Metabolic pathway network of potential biomarkers of related reference articles. Red color for the significantly up-regulated metabolites after TCM treatment in viral pneumonia, blue color for the significantly down-regulated metabolites after TCM treatment in viral pneumonia, black color means controversial significant metabolites after TCM treatment in viral pneumonia, gray color means undetected metabolites

Over the past few years, increasing number of metabolomics studies have focused on the role of viral pneumonia (Table 1) or have studied the metabolic profiles after TCM treatment of viral pneumonia(Tables 2, 3). In this review, recent metabolomics research of viral pneumonia were classified according to the analytical methods used, such as nuclear magnetic resonance (NMR), gas chromatography–mass spectrometry (GC–MS) and liquid chromatography-MS (LC–MS) (Tables 1, 2 and 3). From Table 1 and Fig. 2, we can have a preliminary metabolic understanding of pneumonia infected with H7N9 virus. Serum levels of palmitic acid, erucic acid, and phytal may negatively correlate with the extent of lung inflammation after H7N9 infection. Moreover, the above significant metabolites were related to fatty acid metabolism, which may help to predict a fatal outcome after H7N9 virus infection. Meanwhile, metabolomics is also proved to be a highly sensitive and specific tool for the 90-day prognosis of mortality in H1N1 pneumonia [9, 10].

**Table 1:** Biomarker of viral pneumonia detected by different analytical methods

Altered metabolites	Virus type	Analytical method	Species	Sample material	References
Perturbed serum metabolites at 0, 6, 10, 14, 21 and 28 days post infection Purine metabolism: Xanthine, Hypoxanthine Lipid β-oxidation: l-acetylcamitine, Oleoylcamitine, 3-Methylglutarylcarnitine, Pivaloylcarnitine, Hydroxybutyrylcamitine Sphingolipid metabolism: SM(d18:0/18:1), Glucodylceramide(d18:1/16:0), Sphinganine phosphate, Sphingonine 1-phosphate Fatty acid synthesis: 3-Oxododecanoic acid, 3-Hydroxydodecanoic acid, 5,8-Tetradecadienoic acid 2-Aminooctanoic acid, vinylacetyglycine, hexanoylglycine Phospholipid metabolism: LysoPE(20:1), LysoPC(20:3), LysoPE(18:2), LysoPE(20:2) Tryptophan metabolism: l-kynurenine, 4-(2-Aminophenyl)-2,4-dioxobutanoic acid, 3-Indolepropionic acid, Indoleacetic acid, Indoleacrylic acid, Indoxylsulfuric acid Other metabolic pathways: Tyramine glucuronide, Hippuric acid, Eicosapentaenoic acid, 2-Hydroxy-3-methylbutyric acid, 3-Indole carboxylic acid glucuronide, 12-HETE, 13-HODE, Citric acid, GangliosideA2(D18:1/9Z-18:1), Imidazoleacetic acid, Isohomovanillic acid, Alanylvaline, Tetradecanedioic acid, Pantothenic acid	IFV	LC–MS	Mice	Serum	[58]
Perturbed lung metabolites at 0, 6, 10, 14, 21 and 28 days post infection Purine metabolism: Guanodine, Deoxyadenosine monophosphate, Succinyladenisine, Xanthine, Deoxyinosine, Adenosine monophosphate, Guanosine monophosphate, Adenosine, Diphosphate, Cyclic AMP Pyrimidine metabolism: 5-Thymidylic acid, Thymidine, Deoxycytidine monophosphate, Uridine, Deoxucytidine, Uridine monophosphate, Cytidine monophosphate, Uridine diphosphate Lipid β-oxidation: l-acetylcarnitine, Hydroxybutyrylcarnitine, Propionylcamitine, Butenylcarnitine, Hydroxyhexanoycarnitine, 2-Octenoylcarnitine Sphingolipid metabolism: Sphingosine, Sphinganine Phospholipid catabolism: PC(44:6), PC(32:1), PC(20:1/p-18:0), PC(34:1), LPE(22:4), LPE(18:0) Amino sugar and nucleotide sugar metabolism: UDP-*N*-acetylglucosamine, GDP-l-fucose, d-mannose-1-phosphate Dipeptide: Leucyl-aspartate, Leucyl-glycine, Glycyl-tyrosine, Leucyl-proline, Glycyl-phenylalanine Other metabolic pathways: Tyrosine, 5-Hydroxyindoleacetic acid, Phenylalanine, l-kynurenine, l-leucine, Pyruvic acid, *N*-acetyl-l-aspartic acid, l-2-aminoadipic acid, l-methionine, MG(22:6), *N*-acetylneuraminic acid, Pantothenic acid, Tetrahydroxyperin, *S*-lactoylglutathione, Taurine, Glutaconic acid	IFV	LC–MS	Mice	Lung	[58]
Perturbed BALF metabolites at 0, 6, 10, 14, 21 and 28 days post infection Purine metabolism: Xanthine, Hypoxanthine, Inosine, Guanosine, Cyclic AMP Pyrimidine metabolism: Uric acis, Deoxycytidine, Cytidine, Uridine, Uracil Fatty acid synthesis: 3-Oxotetradecanoic acid, Oleic acid, Palmitic acid, Octadecanedioic acid, Arachidonic acid, Linoleic acid, Docosahexaenoic acid, Tetracosahexaenoic acid, Glutathione metabolism: Oxidized glutathione, Glutathione Fatty acid metabolism: Eicosapentaenoyl ethanolamide, Palmitoleoyl ethanolamide Lipid β-oxidation: Hydroxymyristoylcarnitine, Palmitoylcarnitine, Oleoylcarnitine, l-carnitine, Linoelaidylcarnitine, etc. Phospholipid catabolism: LysoPC(16:1),LysoPC(18:2), LysoPC(20:4), LysoPC(14:0), LysoPC(18:3), LysoPC(20:5), LysoPC(14:1), LysoPE(14:1), LysoPE(22:6), LysoPE(20:4), LysoPE(16:0) Glycerolipid metabolism: MG (22:4), MG(18:4), MG(24:6), Glycerophosphocholine Porphyrin and chlorophyll metabolism: Bilirubin, Biliyerdine Sphingolipid metabolism: Sphingosine, SM (34:1) Phenylalanine metabolism: Phenylalanine, Axetyl-phenylanaline, Hippuric acid Tryptophan metabolism: 3-Hydroxyanthranilic acid, Tryptophan, Indolelactic, 3-Indolepropionic acid Other metabolic pathways: 3-Hydroxyisobutyric acid, Acety-l-leucine, l-leucine, l-valine, Corticosteronr, 3-Furoic acid, Glutaminyl-aspatate, Methionyl-proline	IFV	LC–MS	Mice	BALF	[58]
Upregulate: Acetoacetate, Beta-alanine, Formate, Dimethylamine, Carnitine, Glycine, Gulonic acid, Pentadecane, 2-Amino butanoic acid, Alkane, Quinic acid, Benzoic acid Downregulate: Citrate, Fumarate, 3-Methyl, 2-Isovalerate, Alanine, Tyrosine, Methionine, Histidine, 4-Hydroxybutyrate, Uric acid, Tyrosine, Citric acid, Asparagine, Myoinositol, Lysine, Arabinonic acid, Threonine, Aspartic acid, Threonic acid	H1N1	^1^H–NMR GC–MS	Human	Plasma	[45]
Upregulate: *N*-acetylglucosamine–*N*-acetylgalactosamine, Mevalonolactone, Cystine, Palmiticamide, Caprate (10:0), Laurate (12:0), 2-methylcitratehomocitrate, Diglycerol, Beta-hydroxyisovalerate, Pregnen-dioldisulfate	RSV	UPLC-MS	Human	NPA	[57]
Upregulate: *N*-acetylthreonine, *N*-acetyltyrosine, N6-acetyllysine, Adenine, Azelate (nonanedioate), *N*-acetylvaline, *N*-acetylleucine, Glucoronate, *N*-acetylphenylalanine, *N*-acetylisoleucine, 1-Stearoyl-2-linoleoyl-GPE(18:0/18:2), *N*-acetyltaurine, sulfate, Glycerol, *N*-acetylglutamate, 4-Hydroxyphenyl pyruvate, Glycerol 3-phosphate, *N*-formyl-phenylalanine, Gluconate, *N*-acetylarginine, Folate	RV	UPLC-MS	Human	NPA	[57]
Upregulate: Kanzonol I, Alkaloid A6, Beta-d-glucopyranuronic acid, Protoporphyrinogen IX Downregulate: LysoPC(16:0), LPA(18:1/0:0), LysoPC(18:2), LysoPC(18:1) Palmitic acid, Hydroxyisocaproic acid, Phytanic acid, Erucic acid, Behenic acid, Palmitic amide, Phytal, Caproylcholine, (9S,10S)-10-Hydroxy-9-octadecanoate, 1-Octen-3-yl glucoside, *N*-[(4E,8E)-1,3-Dihydroxyoctadeca-4,8-dien-2-yl] Hexadecanamide, Methyl (9Z)-8′-Oxo-6,8′-diapo-6-carotenoate, and Methyl (9Z)-6′-oxo-6,6′-Diapo-6-carotenoate	H7N9	UPLC-MS	Human	Serum	[9]

NPA, nasopharyngeal aspirate; UPLC-MS, using ultra-performance liquid chromatography-mass spectrometry; 1H-NMR, 1H nuclear magnetic resonance spectroscopy; GC–MS, gas chromatography-mass spectrometry; LC–MS: liquid chromatography–mass spectrometry; TG, triglyceride; LysoPE, lysophosphatidylethanolamines; LysoPC, lysophosphatidylcholines; PE; phosphatidylethanolamines; PC, phosphatidylcholines

**Table 2:** Metabolomics approach on TCMs-derived compounds or aqueous extracts in treatment of viral pneumonia in animal models

Herbs	Compounds/part used	Virus type	Sample material	Detection method	Metabolomics validation (treatment group vs. model group)	References
*Scutellaria baicalensis Georgi*	Baicalein	RSV	HEP-2 Cell	GC–MS	Upregulate: Serine, Aspartic acid, Glutamic acid, Citric acid, Glycine	[12]
			Plasma		Upregulate: Lactic acid, Urea, 1,5-Sorbitan Downregulate: Glycine, Glutamine, d-glucose	
			Urine		Downregulate: Lactic acid, Malic acid, Palmitic acid, Stearic acid	
			BALF	LC–MS	Upregulate: Glutamate, O-octadecenoyl-carnitine, Adenosine, Glucosylceramide, Downregulate: Leukotriene D5	
*Scutellaria baicalensis Georgi*	Root	RSV	Plasma	LC–MS	Upregulate: LysoPC(16:0), LysoPC(18:0), LysoPC(20:4), SM(d18:1/24:1), LysoPC(18:2)	[13]
			Lung		Upregulate: TG(18:1/18:2/18:2), TG(18:1/18:1/18:2), TG(18:1/18:1/18:1), PC(16:0/18:1)	
*Polygonum cuspidatum Sieb. Et Zucc*	Rootstalk and root	RSV	Plasma	LC–MS	Upregulate: LysoPC(14:0), LysoPC(15:0), LysoPC(18:2), LysoPC(17:1), LysoPC(16:0), LysoPC(18:1), LysoPC(18:0), CerP(d13:0/26:3), PC(18:0p/16:0), PC(16:0/18:0), CerP(d15:0/32:3), TG(18:0/18:1/20:1), TG(20:1/18:1/22:0) Pathway: Phospholipid metabolism	[14]
*Flos Lonicerae Japonica and Fructus Forsythiae*	Flower; Fruit	H1N1	Plasma	GC–MS	Downregulate: 3–4-Dihydroxycinnamic acid, Betamannosylglycerate, Glycine, Hydrocinnamic acid Pathway: Galactose metabolism”; “Glycine, serine and threonine metabolism”; “Synthesis and degradation of ketone bodies”	[59]

^1^H-NMR,^1^H nuclear magnetic resonance spectroscopy; GC–MS, gas chromatography-mass spectrometry; LC–MS: liquid chromatography–mass spectrometry. BALF, Bronchoalveolar lavage fluid; RSV, respiratory syncytial virus; HIN1, H1N1 Flu Virus (Swine Flu). TG, triglyceride; LysoPE, lysophosphatidylethanolamines; LysoPC, lysophosphatidylcholines; PE; phosphatidylethanolamines; PC, phosphatidylcholines

**Table 3:** Metabolomics approach on TCM formulas in treatment of viral pneumonia in animal models

Names	Plants or mineral	Part used	Virus type	Sample material	Detection method	Metabolomics validation (treatment group vs. model group)	References
Modified Jiu Wei Qiang Huo decoction (MJWQH)	*Notopterygium incisum Ting ex H. T. Chang*	Root or Rootstalk	H1N1	Serum	LC–MS	Upregulate: l-valine, O-succinyl-l-homoserine, Downregulate: Lauroyl carnitine, Palmitoyl-l carnitine, l-Ornithine, Uric acid, Taurine, l-Leucine, l-Phenylalanine, PGF2α, 20-ethyl-PGE2, Arachidonic acid, Glycerophospho-*N*-arachidonoyl Ethanolamine	[60]
	*Scutellaria baicalensis Georgi*						
	*Astragalus membranaceus (Fisch.) Bunge*						
	*Atractylodes Lancea (Thunb.) DC*						
	*Saposhnikovia divaricata (Trucz.) Schischk.*						
	*Taraxacum mongolicum Hand. Mazz*	Whole plant					
Jinxin oral liquid (JOL)	*Ephedra sinica Stapf*	Stem	RSV	Plasma	GC–MS	Upregulate: Glutamine, Glucose, Arachidonic acid Downregulate: Lactic acid, Urea, 1,5-anhydro-d-sorbitol	[12, 15]
	*Prunus armeniaca L. var. ansuMaxim*	Seed					
				Urine	GC–MS	Downregulate: Lactic acid, Glycine, Stearic acid, Palmitic acid	
	*Gypsum Fibrosum*	CaSO_4_·2H_2_O					
				Spleen	GC–MS	Upregulate: Glutamic acid Downregulate: Proline, Valine, Urea, Glucose, Hypoxanthine	
	*Scutellaria baicalensis Georgi,*	Root or Rootstalk		Plasma	LC–MS	Upregulate: SM(d18:1/24:1), PC(P-20:0/14:0), LysoPC(16:0),LysoPC(18:0), LysoPC(20:4), LysoPC(18:2), LysoPC(18:1), LysoPC(22:6), Phytosphingosine, Downregulate: TG(18:1/18:1/18:2), PE(18:3/19:0)	
	*Polygonum cuspidatum Sieb. etZucc*						
	*Morus alba L.*						
	*Peucedanum praeruptorum Dunn*			Lung	LC–MS	Upregulate: TG(18:1/18:1/18:1), PC(18:2/20:4), TG(18:1/16:0/20:1), PC(22:6/18:2), Choline, LysoPC(18:0), TG(18:1/18:0/20:1), Leucine, LysoPC(16:0), Phytanicacid, Sphinganine, Phenylalanine, LysoPC(18:1), LysoPC(20:4), LysoPC(18:2), LysoPE(16:0)	
	*Lepidium apetalum*	Seed					
Mahuang Xixin Fuzi decoction (MXF)	*Ephedra sinica Stapf*	Stem	H1N1	Feces	LC–MS	Upregulate: Phosphatidylcholine, Pyridoxal, Lysophosphatidylcholine, Succinic acid, Melatonin Downregulate: Phosphatidylethanolamine, Phosphatidylserine, Oxalacetic acid, l-kynurenine	[61]
	*Asarum sieboldii Miq.*	Root or Rootstalk					
	*Aconitum carmichaeli Debx.*						
Pudilan Xiaoyan Oral Liquid (PDL)	*Taraxacum mongolicum Hand*-*Mazz*	Whole plant	H1N1	Lung	GC–MS	Upregulate: Cytidine, Gluconic acid, Methionine, Xanthine, Xanthosine Downregulate: β-hydroxybutyric acid, Glutaric acid, Sucrose, Thymine, Uridine, Pipecolinic acid, Putrescine	[62]
	*Viola yedoensis Makino*						
	*Isatis indigotica Fort*	Root					
	*Scutellaria baicalensis Georgi*	Root					

^1^H-NMR:^1^H nuclear magnetic resonance spectroscopy; GC–MS, gas chromatography-mass spectrometry; LC–MS: liquid chromatography–mass spectrometry; RSV, respiratory syncytial virus; HIN1, H1N1 Flu Virus (Swine Flu). TG, triglyceride; LysoPE, lysophosphatidylethanolamines; LysoPC, lysophosphatidylcholines; PE; phosphatidylethanolamines; PC, phosphatidylcholines

As a novel approach to understanding disease, metabolomics provides a “snapshot” in time of all metabolites present in a biological sample such as plasma, serum, urine, and many other specimens that may be obtained from either patients or experimental models. Recent reports have suggested that metabolomics analysis may provide clinicians with the opportunity to identify new biomarkers for detailed phenotypes and its progression in viral pneumonia (Table 1).

## Evidences supporting the application of metabolomics in TCM

Entering 21st century, TCM as a holistic approach that attempts to balance the body, mind and spirit in individuals with the environment, is getting more and more popular in the whole world [47]. However, the development of TCM also faces severe challenges and suffers from insufficient modern evidences owing to lack of scientific and technologic approaches. Fortunately, the property of metabolomics consists with the holistic thinking of TCM. Metabolomics may beneficially provide an opportunity to clarify the action mechanism of TCM and the meaning of evidence-based TCM by developing the systematic analysis of the metabolites and discovering various biomarkers and perturbed pathways on TCM syndromes or after TCM treatment.

In recent years, metabolomics has been gradually applied to research in the area of TCM and try to highlight the key role of metabolomics to resolve TCM issues. It may scientifically express the meaning of TCM syndromes and the efficacy of TCM treatment [47–49]. For example, based on GC–MS, a research group in China has established a rat model of myocardial ischemia with blood stasis syndrome and qi-yin deficiency syndrome. The endogenous metabolites in plasma were analyzed, and the metabolic profiles between the two TCM syndromes and normal rats were found to be significantly different [50]. A research group carried out a comprehensive analysis of metabolic patterns of typical Jaundice syndrome (JS) and sub-types. They have identified 44 metabolites in JS. The most altered functional pathway was glutamate metabolism, synthesis, and degradation of ketone bodies, alanine and aspartate metabolism (Fig. 2). The results suggested that metabolomics method would be helpful to establishing a suitable model for reasonably evaluating disease syndrome, exploring pathological mechanism of the syndrome, clarifying the relationships between the syndrome and related diseases [51].

Wang et al. evaluated metabolomic characters of the hepatotoxicity induced by alcohol and the intervention effects of Yin Chen Hao Tang (YCHT), a classic traditional Chinese medicine formula composed of *Flos Artemisiae, Gardeniae Jasminoidis, Fructus* and *Radix et Rhizoma Rhei* for treatment of jaundice and liver disorders in China. The greatest difference in metabolic profiling was observed from alcohol-treated rats compared with the YCHT-treated rats [52]. In Table 2, we summarized a kind of potential anti-virus compound-*Baicalein* synthesized from *Scutellaria baicalensis Georgi,* reported to be used in China for treating RSV pneumonia [8]. In Table 3, anti-viral herbs and formulas were selected that have metabolomics evidences in treatment of viral pneumonia. These simple studies above demonstrated that metabonomics, one of the most important systems biology platforms, showed a potential for identifying and characterizing biochemical responses of organism to TCMs. Meanwhile, this strategy offered a practical method for performing intervened assessments of TCM in the future.

## Metabolomics of TCMs in treatment of viral pneumonia

In ancient times, TCMs, as natural products, were successfully used to treat different ailments owing to their enhanced acceptability in human society, better compatibility with the body [8]. TCMs make use of a vast array of medicinal plants, animals and minerals. Medicinal plants (trees, shrubs, grasses or vines), animals (invertebrates, insect, fish, amphibians, reptiles, mammals) and minerals can be used in different forms like active compounds, aqueous extracts, in fresh or powdered form, etc. [53].

Based on the holistic theory, there are five diagnostic methods in TCM, called inspection, auscultation, olfaction, inquiry, and palpation [54]. Diseases are perceived as a disharmony (or imbalance) in the functions or interactions of yin, yang, qi, blood, organs, meridians etc. and of the interaction between the human body and the environment [55]. According to TCM theory, the patterns of bodily disharmony are described in terms of eight major parameters: external and internal, yin and yang, hot and cold, and excess and deficiency. TCM formulas are made up with a relatively complete set of hierarchy principles, the so-called “monarch,” “minister,” “assistant,” and “guide components” [8, 56]. TCM practitioners uphold the principle of making the ancient serve the contemporary, and strive to promote the modernization of TCM by making every effort to carry on the good traditions and practices of Chinese medicine, and promote the innovative development of TCM for health preservation.

Eight published metabolomics & TCM extracts-related articles were studied using different research engines like PubMed, Google, Google-scholar, Science Direct and CNKI. Metabolites in Table 1, including triglycerides (TGs), lysophosphatidylethanolamines (LysoPEs), lysophosphatidylcholines (LysoPCs), phosphatidylethanolamines (PEs), phosphatidylcholines (PCs), ceramides, amino acids and carbohydrates were perturbed in viral pneumonia groups [9, 10, 57, 58]. Metabolomics data (Table 2) suggested that the active compound *Baicalein*, herbs, such as *Scutellaria baicalensis Georgi, Polygonum cuspidatum Sieb. Et Zucc, Flos Lonicerae Japonica and Fructus Forsythiae* were effective to combat viral pneumonia [12–14, 59].

Five published metabolomics & TCM formulas-related articles were listed. 17 TCM-related plant species and 4 TCM formulas (or Chinese patent medicines) were effective to combat viral pneumonia (Table 3). In which, modified Jiu Wei Qiang Huo decoction (MJWQH), Mahuang Xixin Fuzi decoction (MXF) and Pudilan Xiaoyan Oral Liquid (PDL) were effective in treatment of H1N1 pneumonia. Jinxin oral liquid (JOL) is an ancient formula widely used in the treatment of pneumonia and asthma [8]. To investigate the mechanism of the effect of JOL in RSV infected mice, metabolomics approaches based on LC–MS and GC–MS were applied. Samples, such as mice plasma, lungs and spleen were collected. After JOL treatment, compared with the model group, glutamine and glutamic acid were upregulated and lactic acid, urea were downregulated consistently in different types of samples. Most types of lipids were elevated after the TCM treatment.

## Conclusions and future considerations

Metabolomics, as a systemic approach, is a “top-down” strategy to reflect the function of living organisms from the end products of the metabolic network [49]. It will also help to understand the metabolic changes of a complete system under different kinds of physiological and pathological conditions. This property of metabolomics agrees with the holistic thinking of TCMs, suggesting it has the potential to improve our understanding of the theory behind the evidence-based TCM. This review revealed that many of the important information like TCMs preparation, part used, TCMs-derived active compounds, TCMs including aqueous extracts and traditional Chinese medical formulas (or patent medicines) in treatment of viral pneumonia.

From metabolomics research that has been conducted in this review, we can see that all the significant pathways (Fig. 2) in viral pneumonia are related to tricarboxylic acid cycle (TCA). Amino acids are active biomarkers in those pathways, including tryptophan metabolism, alanine, aspartate and glutamate metabolism, arginine and proline metabolism, d-glutamine and d-glutamate metabolism, phenylalanine metabolism, glycine, serine and threonine metabolism, valine, leucine and isoleucine biosynthesis, cysteine and methionine metabolism, vitamin B6 metabolism. Nucleotides, The products of amino acids, also contribute greatly to TCA. Nucleotides metabolism is composed of purine metabolism, pyrimidine metabolism, glutathione metabolism. Based on our previous research, arachidonic acid, fatty acid and lipid metabolism is turning to take the leading role in TCMs treatment of viral pneumonia. Currently, most studies of TCM in treatment of viral pneumonia are untargeted metabolomics and further validations should focus on targeted metabolomics combined with molecular biology technologies.

Metabolomics is going to be a powerful approach to support TCM research in the future. However, the issues and future directions of using metabolomics in TCM studies should be pointed out. The current metabolomics technologies in research on TCM is still in its infancy due to its chemical nature of multi-component mixtures that often possess their own inherent holistic bioactivities. We also highlight the potential role of metabolomics technologies in evidence-based studies of TCM disease-syndrome combination models. Very limited number of metabolomics research provides toxic profile of TCMs, metabolomics toxicity studies should be carried out for TCMs in animal system to establish a safe dose range and specific adverse effect. Further metabolomics-pharmacological research is required to promote the traditional knowledge of TCM and take it to the light of science.
